# Pediatric Water Bead Ingestion: Experience from a Quaternary Care Children's Hospital and a Proposed Management Algorithm

**DOI:** 10.1016/j.jpedcp.2026.200239

**Published:** 2026-08-18

**Authors:** Kacie H. Denton, Madeline van Heukelum, Derek G. Armstrong, Matthew A. Buendia, Maribeth R. Nicholson

**Affiliations:** D. Brent Polk Division of Pediatric Gastroenterology, Hepatology, and Nutrition, Department of Pediatrics, Monroe Carell Jr. Children's Hospital at Vanderbilt, Nashville, TN

**Keywords:** superabsorbent, ultrasound, endoscopy, foreign body, obstruction

## Abstract

**Objective:**

Water bead ingestion has become increasingly common, with variation in management strategies. This study examines the presentation, management, and complications associated with water bead ingestion at a quaternary care children's hospital, situating it within the broader context of the medical literature and proposing a management algorithm.

**Study design:**

This is a case series of pediatric patients who presented to a quaternary care children's emergency department for water bead ingestion from 2015 to 2024.

**Results:**

There were 36 pediatric encounters involving water bead ingestion from 2015 to 2024, with a median age of 2 years (IQR, 1-6), and the majority (n = 28, 78%) were asymptomatic. A total of 52 imaging studies were performed, of which 4 (8%) were abnormal. Endoscopy was performed in 6 (17%) patient encounters, none of which successfully retrieved water beads. In our case series, there were no patients with bowel obstructions or complications.

**Conclusions:**

Best practices for managing children with water bead ingestion remain unclear. Although there were no complications noted in this study, complications due to water bead ingestion do occur, and we support continued efforts to decrease the risk of injury and death associated with water bead ingestions. Upon reviewing our findings and comparing them with current literature, we propose a new algorithm for managing water bead ingestion. This algorithm is intended as a provisional, experience-informed framework to guide management, reduce unnecessary interventions, and standardize care but will require further validation.

Water beads, superabsorbent polymers often marketed as sensory toys, are increasingly recognized as a hazardous ingestion risk in children. These beads can expand upon consumption, and severe outcomes—including small bowel obstruction and death—have been reported.^1^, ^2^, ^3^, ^4^, ^5^ The North American Society for Pediatric Gastroenterology, Hepatology, and Nutrition (NASPGHAN) 2015 guidelines recommend emergent endoscopic removal of ingested water beads within 2 hours of presentation to the emergency department in response to concerning early case reports.^6^ The authors noted, however, that this guidance was based on few reports, and the decision to pursue endoscopy remained at the discretion of the endoscopist.

Since 2015, additional reports detailing ingestions of water beads have been described, although most have focused on patients with complications.^1^, ^2^, ^3^, ^4^, ^5^ Clinical experience, however, suggests that many pediatric patients recover well after ingesting water beads, yet there is a paucity of outcome data in the literature.^7^, ^8^, ^9^ Thus, we aim to identify the presentation, management, and outcomes of patients presenting with water bead ingestion at a large pediatric quaternary care referral center and to compare our results with the broader literature, with the goal of creating an algorithm that balances safety, efficiency, resource utilization, and patient outcomes.

## Methods

### Ethics Statement

This study was reviewed and determined to be exempt by the institutional review board at Vanderbilt University Medical Center.

### Data Collection

This is a case series of water bead ingestions in pediatric patients assessed at Monroe Carell Jr. Children's Hospital at Vanderbilt (MCJCHV) from April 2015, following publication of the NASPGHAN Foreign Body Management guidelines, until April 2024. Patients were identified using the electronic medical record (EMR) via Epic SlicerDicer tool and consult records. SlicerDicer is a reporting tool embedded within the EMR that allows customized searches using data exploration tools to extract the population denominator of interest.^10^ The population of interest was then manually reviewed to identify patients who met the study's inclusion/exclusion criteria, including patients from infancy to 18 years old who presented to the emergency department at MCJCHV with water bead ingestion. Results from SlicerDicer were compared with consult records, as water bead ingestion frequently resulted in a pediatric gastroenterology consultation, to ensure that all patients were captured.

Patient charts were reviewed to obtain demographics, symptoms, imaging, endoscopy characteristics, and outcomes. Interventions, including imaging, admission, and endoscopy, were based on provider preference, as no standard operating procedure was in place during the study period. Data were stored and managed in Research Electronic Data Capture tools hosted at Vanderbilt Health and analyzed using STATA (version 12; StataCorp LLC).^11^^,^^12^ Continuous variables were compared using the Wilcoxon rank-sum test and categorical variables using the Fisher exact test.

## Results

From April 2015 to April 2024, 35 unique patients presented to MCJCHV with water bead(s) ingestion for a total of 36 patient encounters. Analyses were conducted at the encounter level, as each event represented a distinct clinical presentation, evaluation, and management. Of these encounters, 19 (53%) were in children <3 years old, with a median age of 2 years (IQR 1-6) and an overall range of 0-16 years. There were 22 (61%) encounters with a female patient. Additional demographics are summarized in Table I.

**Table I:** Demographics of patient encounters with water bead ingestion

Encounter characteristics	n = 36, n (%)
Age in years, median (IQR)	2 (1-6)
Female sex, n (%)	22 (61%)
Past medical history	
Autism	6 (17)
Developmental delay	8 (22)
Other	13 (37)
Event	
Ingestion witnessed	11 (31)
Intentional ingestion	7 (19)
Type of water bead	
Orbeez	12 (33)
Toy	8 (22)
Stress ball	6 (17)
Ice pack	3 (8)
Symptomatic	
Nausea/vomiting	0
Abdominal pain	4 (11)
Abdominal distention	1 (3)
Fever	0
Decreased appetite	1 (3)
Fussiness	2 (6)
Throat clearing	1 (3)
Constipation	1 (3)
Physical examination findings	
Abdominal tenderness	1 (3)
Abdominal distention	3 (8)
Guarding	1 (3)
Imaging	
XR performed; XR abnormal	33 (92); 3 (9)
Ultrasound performed; XR abnormal	18 (50); 1 (6)
CT performed; CT abnormal	1 (3), 0
Endoscopy	
Endoscopy performed	6 (17)
Endoscopy abnormal	0
Surgical consult	10 (28)
Disposition	
Discharged from ED	19 (53)
Discharged after EGD, no admission	2 (6)
Admission for observation and EGD	4 (11)
Admission for observation without EGD	11 (31)

*ED*, emergency department.

Many of these encounters included patients with behavioral or developmental conditions; 6 (17%) had autism, 8 (23%) had developmental delay, and 1 (3%) had pica. Of the 36 ingestion events, 25 (69%) were unwitnessed and 29 (81%) were reported as unintentional. The type of water beads varied, with the name brand Orbeez (Spin Master Corporation) being identified as the most common (33%). Other water beads were noted to come from a toy, stress ball, or ice pack. There were 27 (75%) encounters for which the time of ingestion was reported in the EMR, with a median (IQR) of 2 (1-4) hours from ingestion to ED presentation.

Most patients were asymptomatic at presentation (n = 28, 77%). Symptomatic children did not differ from asymptomatic children by age (*P* = .24) or gender (*P* = .63). Four of the 8 (50%) encounters with symptomatic patients reported abdominal pain. Additional symptoms included abdominal distention (n = 1), fussiness (n = 3), throat clearing (n = 1), and constipation (n = 1). On physical examination, 3 (38%) encounters with symptomatic patients exhibited abdominal distension; 1 (13%) also had abdominal tenderness and guarding. All patient encounters with abnormal physical examination findings involved children who also reported symptoms.

Imaging modalities used during these patient encounters included abdominal radiograph (XR), transabdominal ultrasound (US), and computed tomography (CT). XR was the primary imaging modality, used in 33 (92%) patient encounters. In those with a reported time of ingestion, the hours from ingestion to XR ranged from 1 to 36, with a median (IQR) of 5 (2-6) hours. There were 30 (91%) encounters with normal XR, with 2 (6%) concerning for a possible foreign body, and 1 (3%) with loops of distended bowel. In one-half (50%) of the patient encounters, an US was completed; of these, in 1 patient, water beads were identified within the small intestine; otherwise, all were normal. In encounters with a known time of ingestion, the hours from ingestion to US ranged from 2 to 41, with a median (IQR) of 7 (5-10) hours. A single CT was performed 6 hours after initial ingestion, and the findings were normal.

Of the 8 symptomatic patient encounters, all had normal XR, 4 (50%) had normal US, 2 (25%) had normal esophagogastroduodenoscopy (EGD), and 7 (88%) were admitted for observation. An encounter involving a symptomatic child was more likely to result in a hospitalization than those involving an asymptomatic child (88% admitted vs 29% admitted, *P* = .005).

There were 6 (17%) patient encounters in which patients underwent an EGD. The median (IQR) age of patients during these encounters was 5 (2-12) years. There were no statistically significant differences in age, gender, or symptom presence between those who underwent EGD and those who did not, although analysis is limited by sample size. Two (33%) of these patients reported symptoms at the time of encounter. Three (50%) of these patient encounters included a time of ingestion, with time to EGD from ingestion ranging from 4 to 18 hours. In all EGDs, no water beads were visualized, and therefore, no water beads were retrieved. Notably, none of the endoscopic procedures were performed within 2 hours of ingestion, limiting assessment of early endoscopic retrieval efficacy. All patients who underwent EGD were discharged after the procedure, including the 2 symptomatic patients who had been admitted for observation.

Regarding disposition, 21 (58%) encounters involved patients discharged from the ED or immediately after EGD, while 15 (42%) encounters included patients admitted for observation. Of those admitted for observation, 7 (47%) patients endorsed symptoms, and 3 (20%) patients had XR concerning for a foreign body. Surgery was consulted for 3 (8%) patient encounters, all of whom were admitted for observation. Outside of supportive care, including intravenous fluids, bowel rest, or initiation of a daily bowel regimen, no other medical management was performed during admission.

There were no patients in our case series who were diagnosed with a small bowel obstruction or required surgical intervention. No patients developed complications, and no subsequent encounters associated with water bead ingestion were documented to suggest further complications.

## Discussion

In our quaternary care children's hospital, we identified 36 cases of water bead ingestion over a 9-year period, from 2015 to 2024, none of which resulted in complications. This is similar to a prior case series from 2011 to 2016, which examined 110 patients aged 19 years or younger who had called the Texas Poison Control Center for water bead ingestion. The study found that many were managed at home, without any known complications.^13^ A 10-year retrospective analysis of water bead ingestion in children ages 2 through 15 years was performed in Turkey.^8^ The results described all 21 patients as asymptomatic with no complications. Additionally, a Virginia regional poison center reviewed 217 cases of water bead ingestions; none of which had abnormal imaging, required admission or procedural intervention, or developed bowel obstruction.^7^

However, there are reports of morbidity and mortality associated with water bead ingestions. A literature review published in 2022 described 43 cases of bowel obstruction and 2 deaths following the ingestion of water beads.^3^ The report synthesized data from 20 case reports and 4 case series. Among children for whom individual ages were reported, the median age of children was 15 months (IQR, 1-18). There are important differences between the children who had complications and those described in our case series. Children in our case series were older, with a median age of 2 years (IQR, 1-6) vs 15 months (IQR, 1-18). Children with complications were also more likely to have abnormal imaging results; 31 (72%) children underwent XR, all of which demonstrated air-fluid levels and/or bowel dilatation. In contrast, our study revealed 33 (92%) encounters including imaging, and only 1 XR had concerns for obstruction. In addition to the previously published literature review, we identified 31 additional cases of bowel obstruction following water bead ingestion reported in the literature, including 1 fatality (Table II).^5^^,^^14^, ^15^, ^16^, ^17^, ^18^, ^19^, ^20^, ^21^, ^22^, ^23^, ^24^, ^25^, ^26^, ^27^ One abstract utilized data from the National Poison Data System, raising the possibility of case overlap with a prior report.^27^ Among these 31 cases, most patients were younger than 2 years, 19 (61%) patients required exploratory laparotomy, and only 3 (10%) patients underwent successful endoscopic retrieval of water bead(s).

**Table II:** Recently reported cases of bowel obstruction secondary to water bead ingestion

References	Year published (Years of study)	Number of patients	Age	Symptoms (%)	Imaging, % with imaging (% with abnormal imaging)	Endoscopy performed (%)	Removal of beads with endoscopy (%)	Surgical intervention (%)	Mortality
Kim et al^14^	2020	1	12 mo	Yes	XR (0%), US (100%)	Yes	Yes	No	No
Mullens et al^15^	2021	1	10 mo	Yes	XR (100%), US (100%), CT (100%)	No	No	Yes	No
Gaines et al^16^	2022	1	2 y	Yes	XR (100%)	Yes	No	Yes	No
Jung et al^18^	2022	2	10 mo 16 mo	Yes (100%)	XR 100% (100%), CT 100% (100%)	No	No	Yes (100%)	No
Castro et al^17^	2023	1	10 mo	Yes	XR (100%)	No	No	Yes	No
Govindarajan et al^19^	2023	2	1 y 2 y	Yes (100%)	XR 100% (100%)	No	No	Yes (100%)	No
Ranjithatharsini et al^25^	2023	1	1 y	Yes	XR (100%), US (100%), CT (100%)	No	No	Yes	No
Van Horn et al^26^	2023	1	2 y	Yes	NA	No	No	Yes	No
Awolaran et al^20^	2024	1	"infant"	Yes	NA	Yes	No	Yes	No
Haider et al^21^	2024	1	19 y	Yes	CT (100%)	Yes	Yes	No	No
Nicodemus et al^24^	2024	1	14 mo	Yes	CT (100%)	No	No	Yes	No
Wahl et al^27^	2024 (2019-2022)	14	0-5 y	NA	XR 42.9% (28.6%), US 14.2% (14.2%)	Yes (71.4%)	NA	Yes (35.7%)	No
Bollettini et al^5^	2025	2	11 mo 11 mo	Yes (100%)	XR 100% (100%), US 100% (100%)	Yes (50%)	Yes (50%)	Yes (50%)	No
Haugen et al^22^	2025	1	13 mo	Yes	XR (100%) US (100%) CT (100%)	No	No	Yes	No
Makele et al^23^	2025	1	2 y	Yes	XR (100%), US (100%)	No	No	Yes	No

Although our report suggests that most patients do well after water bead ingestion, complications reported with water beads strongly support the need to further reduce the risk of injury or death in pediatric patients. Thus, we endorse the recent consumer product safety standard released by the United States Consumer Product Safety Commission.^28^ The new standard for water beads sets a maximum expansion size limit to prevent them from becoming large enough to cause a blockage, establishes limits on the amount of allowable acylamide to reduce toxicity risks, and requires strongly worded, easily seen warning labels to caution customers. Water beads encompass a heterogeneous group of superabsorbent polymer products with variable physical properties and clinical risk profiles. Dehydrated water beads, which would not be at maximum size at ingestion, may warrant added concern due to their ability to expand significantly to an unknown size inside the gastrointestinal tract. In addition to mechanical complications, water beads may also pose a risk of neurotoxicity due to the presence of acylamide, which can manifest as irritability, altered mental status, tremor, or weakness. Acylamide toxicity is related to prolonged water bead retention and warrants recognition.^22^^,^^28^

It remains uncertain as to the best imaging practices in patients after ingestion of water beads. In the NASPGHAN 2015 guidance, XR studies were deemed unlikely to be helpful, related to the radiolucent nature of water beads.^6^ In addition, contrast studies were not recommended due to concern for their ability to delay and complicate plans for endoscopic removal. Since that time, multiple studies have described imaging modalities used with water bead ingestions.

Two-view abdominal XRs are often used as a screening tool to assess signs of bowel obstruction, and there are case reports with radiologic evidence of bowel obstruction due to water beads on XR.^1^^,^^15^^,^^18^^,^^19^^,^^23^^,^^29^ However, one cannot rely solely on an XR to confirm bowel obstruction, as a few symptomatic cases of bowel obstruction from water bead ingestion had a normal XR.^5^^,^^14^^,^^24^

US has emerged as a potentially valuable tool to visualize ingested water beads. Multiple cases have demonstrated the ability of US to identify water beads in the stomach and small bowel.^14^^,^^22^^,^^30^ Caré et al noted visualization of water beads in 80% of patients receiving an US. However, US led to a bowel obstruction diagnosis in less than one-half of their cases of bowel obstruction and. thus, is not a reliable test to diagnose bowel obstruction.^3^ In our report, water beads were identified in only 1 (6%) of 18 patient encounters where US was performed. The reasons for the differences at our center and others are unclear but may relate to the size of water beads or radiologic technique. US with water-soluble contrast may play a role in select cases, particularly in detecting more proximal intestinal obstruction, but data to support this are lacking, and its use must be balanced against potential delays in endoscopic or surgical intervention. Although US cannot consistently diagnose bowel obstruction, it may aid in operative planning for patients with a known obstruction or identify beads within the stomach that may benefit from endoscopic removal.

CT is rarely used in water bead ingestions. In our case series, only 1 patient had a CT, which was unremarkable. Some case reports have described the use of CT; some clearly identified water beads, whereas others did not.^1^^,^^3^^,^^24^^,^^31^ CT led to the diagnosis of bowel obstructions in these cases, which is the gold standard imaging modality for identifying bowel obstructions.

Current NASPGHAN guidelines recommend early endoscopy in cases involving high-risk or potentially obstructive foreign bodies, with emphasis on emergent endoscopy in the case of superabsorbent objects such as water beads.^6^

In our case series, EGD was performed in 6 (17%) patient encounters, and water beads were identified in none. Notably, in patients with a known time of ingestion, zero (0/3) underwent endoscopy within the first 2 hours postingestion. This limits assessment of whether water beads may have been retrievable from the upper gastrointestinal tract earlier after ingestion, which is a critical limitation.

Data are limited regarding the use of endoscopy in presentations of water bead ingestion, but there are reports of successful retrievals. The literature review by Caré et al identified 2 cases in which endoscopic removal of water beads was performed successfully.^3^^,^^32^^,^^33^ In 1 case, water beads were identified in the duodenum, where they were broken down with a grasper and suctioned.^32^ In the second case, duodenal water beads were crushed and removed with forceps.^33^ Three other cases outside of this literature review have described endoscopic removal of water beads.^5^^,^^14^^,^^21^ Of these, water beads were retrieved via crushing and suctioning in the duodenum or via use of a retrieval net in the stomach. Interestingly, one of these cases reported an ingestion of 200 to 300 water beads, where not all beads were retrieved, with the patient being able to pass the remaining beads with the use of polyethylene glycol 3350.^21^ Notably, water beads can pass rapidly through the stomach and into the small intestine. Their hydrophilic surface and gel-like consistency likely facilitate swift transit, limiting the window for endoscopic retrieval. Additionally, endoscopy carries inherent risks that must be considered.

The NASPGHAN guidelines for managing water bead ingestion were last updated in 2015.^6^ However, alternative clinical pathways are also available. The Children's Hospital of Philadelphia's water bead ingestion recommendations include consulting gastroenterology and surgery if the patient is symptomatic and considering endoscopic removal.^34^ One recently published article recommends clinical observation and US for asymptomatic patients younger than 2 years.^5^ For symptomatic patients, the authors suggest using US and upper gastrointestinal series with fluoroscopy to localize bowel obstruction, followed by either endoscopy or exploratory laparotomy. Fluoroscopic studies typically require oral contrast, which may not be advisable in patients who may require urgent procedures under anesthesia. Another recent study examined the expansion of water beads in various fluids and proposed management strategies based on its findings.^35^ They recommended considering the dry size of the bead when evaluating for complications, with those >3 mm being the highest risk of obstruction. However, applying these recommendations may be challenging in practice, as limited information is often available on the quality of water beads at the time of encounter.

At our center, we have observed notable variability in managing water bead ingestions, reflecting the lack of standardized guidelines. Upon reviewing our data and the current literature, we propose a new algorithm for managing pediatric patients presenting with water bead ingestion (Figure). This pathway considers the patient's age and the time of ingestion to improve the yield of EGD. Risk factors associated with water bead ingestion include a patient age less than 2 years old and the time of ingestion to presentation being less than 2 hours. Patients who are asymptomatic with no risk factors can be safely discharged with strict return precautions. If an asymptomatic patient has risk factors, we recommend obtaining US. If water beads are visualized in the stomach, the patient should undergo urgent endoscopy to attempt retrieval of water beads. If no water beads are visualized and the patient is less than 2 years old, we recommend observation. Symptomatic patients, regardless of risk factors, should have a 2-view XR to assess for obstruction. If there is evidence of bowel obstruction, we recommend consulting surgery. Symptomatic patients without evidence of obstruction, however, should be observed for 24 hours. Our approach prioritizes clinical concern for obstruction over localization of ingested beads in symptomatic patients, as symptoms suggest distal progression where endoscopic retrieval is less likely to be beneficial and where further imaging, such as US, may not alter immediate management. Further details are discussed in the algorithm.

**Figure:**
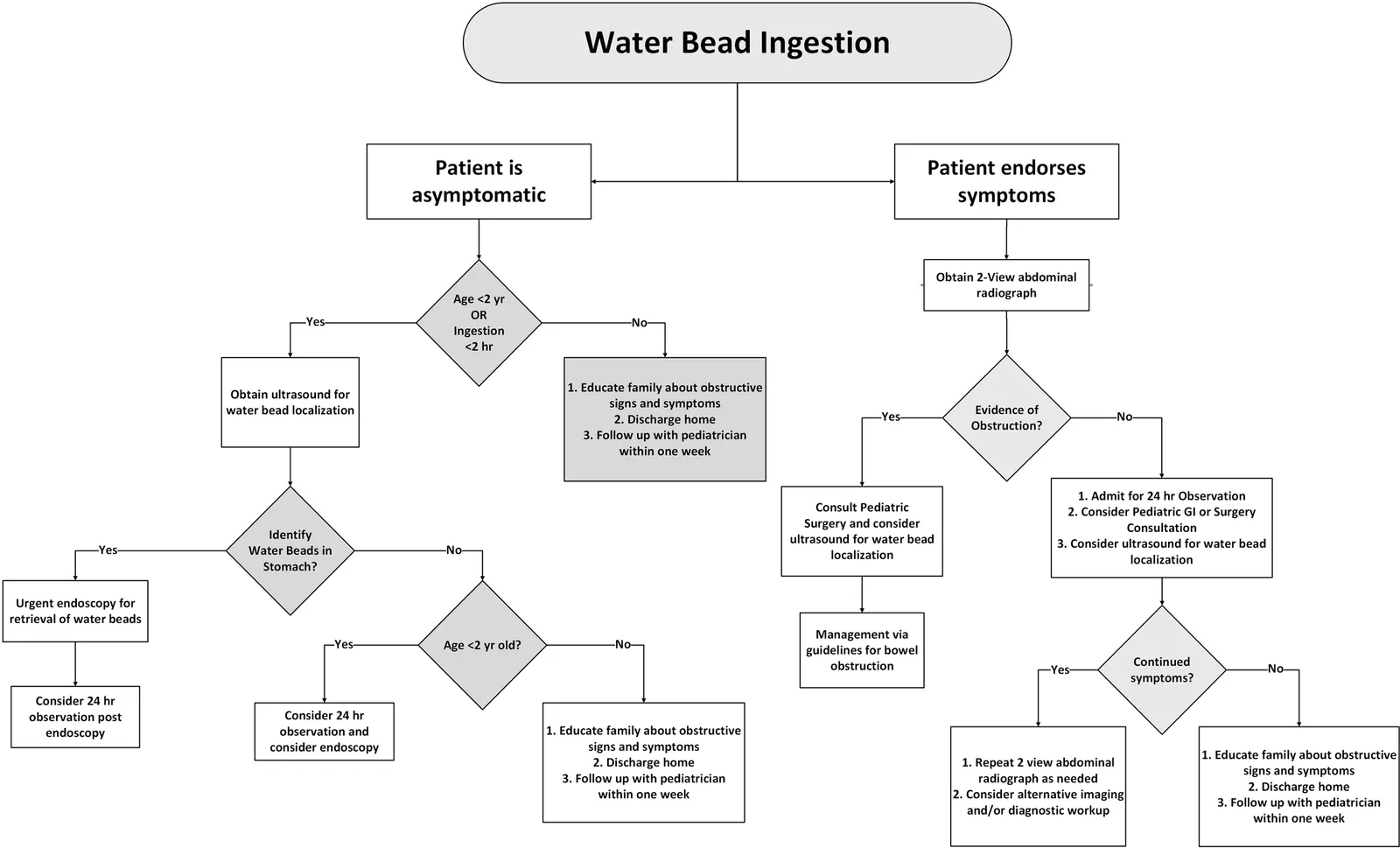
Suggested management of pediatric water bead ingestion.

With this algorithm, we aim to establish a consistent management protocol and reduce unnecessary interventions and hospitalizations, while remaining appropriately cautious of potentially negative outcomes. A few caveats should be mentioned when utilizing our algorithm. As most bowel obstruction cases secondary to water bead ingestion reported in the literature have occurred in patients younger than 2 years, we have taken this into consideration in our management approach. Provider suspicion of alternative complicating factors, such as patient size and intestinal anatomy, should be considered. The timing of endoscopic intervention in our article was informed by existing NASPGHAN guidance, which recommends prompt removal of water beads. This has been termed as emergent endoscopic evaluation. However, we recognize that in clinical practice, several factors influence the feasibility of this timing, limiting the opportunity for early endoscopic retrieval before transit beyond the stomach. Therefore, the emphasis on early endoscopy should be interpreted as a call for timely intervention in select high-risk patients when resources are available. We recognize that other emergent procedures, such as endoscopy for button battery ingestion, carry a significantly higher risk than water beads. Thus, prioritization within the medical setting needs to be carefully considered and may vary by center and resources. There are also data to suggest that the number of water beads ingested could be directly correlated with the degree of severity.^9^ However, other studies show that bowel obstruction can occur with as few as one water bead, negating this correlation.^3^ Thus, we did not include the number of water beads in our algorithm. The size of water bead expansion may also be critical, as highlighted in the recent Consumer Product Safety Commission safety standards.^28^ However, that information is not widely reported and, therefore, is excluded from our current algorithm. Our suggested algorithm remains provisional and informed by both experience and available literature to guide clinical decision-making but will require further validation before widespread adoption.

This study has several limitations. The small sample size limits the extent to which our findings can be generalized. Thus, we have considered our study in the context of additional published works. The absence of patients receiving an endoscopy within 2 hours of ingestion further restricts our ability to evaluate early retrieval of water beads. Finally, it is possible that patients discharged home with water bead ingestion had a later complication not identified at our children's hospital. Given that MCJCHV is a large quaternary care referral center with a wide catchment, and these patients were seen here for an initial evaluation, it is highly unlikely, although possible, that they would not have returned if symptoms or complications had developed.

In conclusion, our study offers important insights into the presentation, evaluation, and management of water bead ingestions in children. By synthesizing our findings with existing literature, we propose a practical, experience-informed algorithm that may serve as a starting point for future efforts to standardize care and guide clinical decision-making. Although no adverse events were observed in our pediatric case series, rare but serious complications, including bowel obstruction and death, have been reported, underscoring the importance of continued vigilance when evaluating pediatric patients with suspected water bead ingestion. As water beads remain widely available, future prospective studies with larger, more diverse cohorts will be essential to validate and refine our proposed management approach, enhance risk stratification, and ultimately optimize patient safety and outcomes.

## CRediT authorship contribution statement

**Kacie H. Denton:** Writing – review & editing, Writing – original draft, Visualization, Validation, Resources, Project administration, Methodology, Investigation, Formal analysis, Data curation. **Madeline van Heukelum:** Writing – review & editing, Writing – original draft, Resources, Data curation. **Derek G. Armstrong:** Writing – review & editing, Data curation, Conceptualization. **Matthew A. Buendia:** Writing – review & editing, Data curation, Conceptualization. **Maribeth R. Nicholson:** Writing – review & editing, Writing – original draft, Visualization, Validation, Supervision, Software, Resources, Project administration, Methodology, Investigation, Funding acquisition, Formal analysis, Data curation, Conceptualization.

## Declaration of Competing Interest

This work was supported by a grant funding for REDCap (UL1 TR000445 from 10.13039/100006108NCATS/NIH). This work was also supported by the 10.13039/100000002National Institutes of Health (NIH) [K23AI156132], granted to the corresponding author, M.R.N. The sponsor was not involved in study design, collection, analysis, and interpretation of the data, the writing of the report, and the decision to submit the paper for publication. All other authors declare no conflicts of interest. The first draft of the manuscript was written by K.H.D., M.v.H., and M.R.N. There was no form of payment given to anyone to produce the manuscript.
